# Speed of structured light pulses in free space

**DOI:** 10.1038/s41598-019-54921-5

**Published:** 2019-12-04

**Authors:** N. I. Petrov

**Affiliations:** Scientific and Technological Center of Unique Instrumentation of Russian Academy of Sciences, Moscow, 117342 Russia

**Keywords:** Slow light, Terahertz optics

## Abstract

A plane monochromatic wave propagates in vacuum at the velocity *c*. However, wave packets limited in space and time are used to transmit energy and information. Here it has been shown based on the wave approach that the on-axis part of the pulsed beams propagates in free space at a variable speed, exhibiting both subluminal and superluminal behaviours in the region close to the source, and their velocity approaches the value of *c* with distance. Although the pulse can travel over small distances faster than the speed of light in vacuum, the average on-axis velocity, which is estimated by the arrival time of the pulse at distances *z* ≫ *l*_*d*_ (*l*_*d*_ is the Rayleigh diffraction range) and *z* > *cτ* (*τ* is the pulse width) is less than *c*. The total pulsed beam propagates at a constant subluminal velocity over the whole distance. The mutual influence of the spatial distribution of radiation and the temporal shape of the pulse during nonparaxial propagation in vacuum is studied. It is found that the decrease in the width of the incident beam and the increase in the central wavelength of the pulse lead to a decrease in the propagation velocity of the wave packet.

## Introduction

The pulse wave packet propagation effects are of considerable interest because of their significance from fundamental and application aspects such as optical communication, information science, digital holography, and image processing.

Spatially and temporally localized beams are of interest since they describe the fields associated with dielectric antennae, laser optical systems and other sources emitting focused radiation more realistically than plane waves. It is known that the spatiotemporal shape of these wave-packets is modified during propagation due to the effects of non-stationary diffraction. One of the reasons leading to the transformation of the space-time radiation structure is the interconnected diffraction transformation of the frequency and angular spectra. Many works have been devoted to the study of the propagation of radiation pulses in various media^1–5^. Non-stationary diffraction effects arising in the propagation of electromagnetic pulses in free space are considered in^2^. In^3,4^, the problem of diffraction of a pulsed Gaussian beam in vacuum is solved in the paraxial approximation, and the relationship between the spatial and temporal characteristics of the beam is investigated on the basis of the spectral approach. In^5^ an approach to the description of the non-stationary diffraction of extremely short pulses based on a generalization of the stationary Sommerfeld diffraction theory is developed.

In recent years, the study of the superluminal and subluminal propagation of ultrashort pulses in free space has been of particular interest. The speed of light propagation in vacuum is one of the fundamental characteristics of electromagnetic waves. A plane monochromatic wave propagates in vacuum at velocity *c*. However, wave packets limited in space and time are used to transmit energy and information. The wave packet propagates at a group velocity that is different from the velocity of individual harmonic components. The phase and group velocities of light pulses can differ significantly in dispersive media such as cold atomic clouds^6^, and atomic vapours^7–9^. In^10^ it was demonstrated experimentally and theoretically the possibility of superluminal propagation of the pulse maximum in an amplifying medium. The superluminal effects were also observed in vacuum. The superluminal group velocity of optical Bessel beam pulses has been experimentally demonstrated in several works^11–16^. Direct measurements of the spatio-temporal electric field of Bessel-X-type pulses generated by a refractive axicon were carried out in^14,15^. The pulsed beam given by the zeroth-order Bessel beam with a Gaussian temporal profile was experimentally studied with fs-range resolution in^17,18^. Recently, non-diffracting optical 2D space-time wave packets in free space were considered^19–21^. Arbitrary group velocities are observed above or below the speed of light in vacuum by modulating the spatio-temporal spectrum.

The effect of the transverse spatial structure of a light beam on its propagation velocity was experimentally discovered in^22^. The effect was explained by the delay of peripheral regions of the beam using the geometric optics approach. However, this approach is insufficient, and a rigorous analysis of the problem is possible only within the framework of wave optics, taking into account nonstationary diffraction effects. It was shown in^23^ that the slowing down of light also depends on the magnitude of the orbital momentum of the beam. The group velocities manifested by Laguerre-Gauss (LG) modes in vacuum were investigated, and the subluminal effects arising from the twisted nature of the optical phase front were observed and explained in paraxial approximations. However, LG functions are not the solutions of the Helmholtz wave equation in free space, and they can be considered as the modes in vacuum only in paraxial approximation. It was noted in^24^, that there is a discrepancy between the theoretical results of^23^ and the well-known results for the simple case of Laguerre-Gauss modes. As clarified in^25^, the discrepancy in group velocities is due to the distinction between Laguerre-Gauss modes and hypergeometric-Gauss modes, which were used in the experiment and in the theoretical analysis. A reduction in the group velocity below the value of *c* for certain Bessel beam pulses was considered theoretically in^26^. An optical buffer in free space that would provide delays on the order of 100 ps over a 1 cm length was proposed. However, as shown in^27^, the practical feasibility of this optical buffer is questionable. It was explained in^28^, that a structured beam acts as a natural cylindrical waveguide as the beam propagates in free space or in dielectric media, and an optical buffer may be possible in the future with advances in photonics technology. In^29^, the group velocity of the LG beam (not pulse) in free space was calculated in the paraxial regime. In^30^, it was shown that the addition of orbital angular momentum (OAM) reduces the delay (accelerates) with respect to the same beam with no OAM. The results are explained using a geometrical ray-tracing approach. Note that the pulsed beams were not considered in these works. Of particular interest were propagation-invariant localized pulsed waves that exhibit non-diffracting non-spreading propagation over a large distance^31^.

In this paper, the theoretical analysis of the nonparaxial propagation of vortex pulsed beams in free space in the framework of the wave approach is carried out, taking into account the non-stationary effects of diffraction. The mutual influence of the spatial distribution of the incident radiation and the temporal shape of the pulse during vacuum propagation is studied. In particular, the subluminal effects arising from the spatial localization of the pulse beam are revealed. The smaller the beam radius is, the slower the propagation speed. A strong change in the pulse shape as a consequence of nonstationary diffraction is demonstrated for tightly focused beams. Spatial modes with azimuthal and radial indices are proposed for the simulation of pulsed beam propagation in free space. Mode representation provides physical insight and computational simplification in the analysis of pulsed beams in free space. The influence of the spatial limitation of the pulse beam on the propagation velocity is studied. It is shown that there is a significant difference between the axial velocity (the velocity measured along the propagation axis at one point of the beam cross-section) and the total pulse beam velocity (when the receiver captures the full cross-section). The on-axis velocity exhibits both superluminal and subluminal behaviours along the propagation distance, whereas the total cross-section velocity is subluminal over the whole distance.

The results of this work extend the known results of the propagation of pulsed beams in free space and can be applied in many areas of optics and photonics, such as optical communication, temporal imaging, and supercontinuum generation.

## Results

First, we find the evolution of each spectral component of the spatiotemporal incident pulse. Then the inverse Fourier transform gives an expression for the electric field in the time domain. It is known that the non-diffracting Bessel beams are the solutions of the Helmholtz wave equation^32,33^. They can be considered as the modal solutions with azimuthal indices in free space. However, Bessel beams have infinite transverse sizes and require infinite power. In practice, quasi-Bessel beams of limited transverse dimensions generated by an axicon or conical lens are used. These beams exhibit no diffraction over a limited propagation distance^32,33^. There are also modal solutions of finite transverse size with discrete azimuthal and radial indices similar to modal solutions in cylindrical waveguides (see section Methods for supporting content). The transverse field profiles of these solutions remain invariant along the effective depth of field. Note that these solutions form a complete set of mutually orthogonal functions in a given interval [0, *R*_0_]. Hence, any field in the initial plane *z* = 0 can be decomposed into these modal solutions.

Wave propagation is characterized by various velocities: the phase, group, signal envelope amplitude, and energy. The velocities for the pulse amplitude, pulse centre of gravity, and pulse energy flow can be considered for pulse beams: ${v}_{m}=z/{t}_{m}$, ${v}_{c.g.}=\frac{z}{{T}_{c.g.}}$ and ${v}_{E}=\frac{S}{w}$, where *z* is the propagation distance (detection plane), *t*_m_ is the arrival time of the pulse amplitude, *T*_c.g._ is the arrival time of the pulse centre of gravity, *S* is the Poynting vector, and *w* is the electromagnetic energy density.

The pulse velocity can be determined from Eq. (25) (see section Methods for supporting content). It follows from this that both the group and phase velocities of the modes contribute to the resulting pulse velocity if only an axis part of the beam cross-section is recorded. This means that the velocity of the pulse beam depends on the size of the receiver aperture.

The energy flow velocity depends only on the group velocities of the modes; thus, the averaged energy flow velocity ${v}_{E}$ of a pulsed beam is always subluminal, i.e., ${v}_{E} < c$. In addition, the instantaneous (local) velocity of propagation can be defined as ${v}_{ins}=\frac{dz}{dt}$ at different distances along the axis of propagation.

The arrival time can be determined both for a given beam cross-section point and for the entire beam, i.e., by averaging over the cross-section.

The arrival time of the pulse centre of gravity, which is determined by ${t}_{c.g.}=\bar{t}(\rho ,z)=\frac{\int I(\rho ,z,t)tdt}{\int I(\rho ,z,t)dt}$, is different for various points of the beam cross-section. The on-axis arrival time is equal to ${T}_{ar}^{axis}=\bar{t}(0,z)$. If the total cross-section of the pulse beam is recorded, then the arrival time is determined by averaging over the entire cross-section: ${T}_{ar}^{tot}=\langle t(z)\rangle =\frac{{\iint }^{}I(\rho ,z,t)\rho d\rho tdt}{{\iint }^{}I(\rho ,z,t)\rho d\rho dt}$.

The spatiotemporal shape of wave packets is modified during propagation due to the nonstationary effects of diffraction and interference of modes. A strong change in the pulse shape occurs when the beam width and pulse duration decrease. Note that the width of the beam in front of the pulse varies less than in the tail part during the propagation. This indicates that the frontal part (high frequencies) of the pulses is less affected by diffraction.

Although the simulation can be performed using the integral representation (see section Methods for supporting content), the simulation results obtained using the modal approach are presented below. The mode representation provides a simplification of calculations in the analysis of pulsed beams in free space.

**Figure 1:**
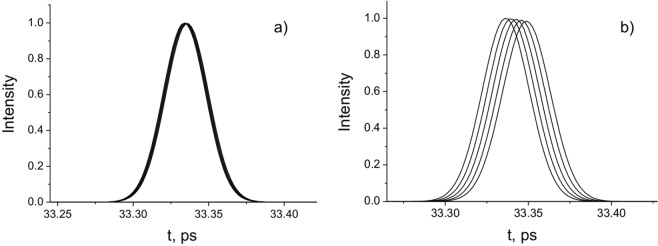
Pulse intensities at the distances *z*_k_ = 10, 20, 30, 40, 50 mm in the coordinate system with time delay $t-\frac{({z}_{k}-{z}_{1})}{c}$. *τ* = 20 fs; (**a**) *a*_0_ = 100 μm is the radius of Gaussian distribution; *w*_*B*_ = 50 μm is the central peak width of the Bessel function; (**b**) *a*_0_ = 100 μm; *w*_*B*_ = 20 μm.

In Fig. 1 the pulse intensities at different distances *z*_k_ are presented. In Fig. 1a,b, the Bessel-Gauss (BG) pulses are presented in an offset time scale $t-\frac{({z}_{k}-{z}_{1})}{c}$, where *z*_*k*_ is the distance, at which the pulse is recorded. It can be seen that the pulses have the same shape at different distances, but they are offset relative to each other. These shifts originate from differences in the time of arrival of the pulse amplitude. This means that the considered pulsed beam experiences subluminal propagation. As the width of the incident beam decreases, the time shift of the amplitude position between the considered pulse and plane-wave propagating at velocity *c* increases.

**Figure 2:**
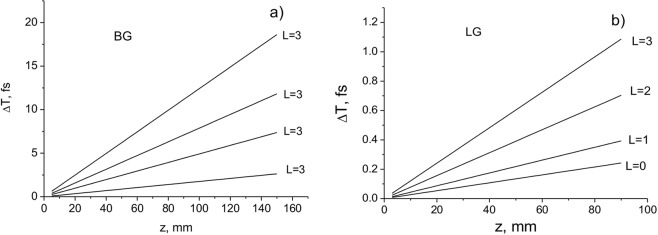
Delays in arrival time as a function of distance *z* for the pulsed BG (**a**) and LG (**b**) beams: *a*_0_ = 100 μm; *w*_*B*_ = 100 μm; *w*_0_ = 100 μm; *λ* = 795 nm.

In Fig. 2, the delay times of the pulsed BG and LG beams (the arrival time of the pulse centre of gravity compared to light in vacuum) $\Delta T={T}_{ar}^{tot}-{T}_{0}$, where ${T}_{0}=\frac{z}{c}$, depending on the distance *z* are presented for different values of OAM. It is shown that the delays are higher for pulsed BG beams and increase with increasing OAM.

**Figure 3:**
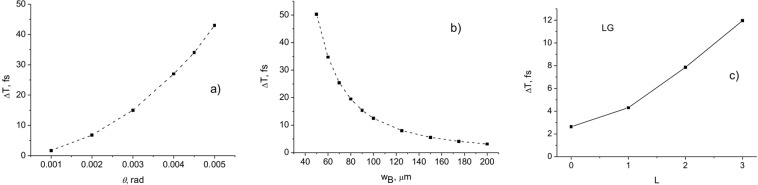
Arrival time delays as a function of the cone angle *θ* (**a**), central peak width *w*_*B*_ (**b**), and orbital angular momentum *L* (**c**) relative to the plane-wave case. The beam parameters are: (**a**,**b**) pulsed BG beam with *τ* = 100 fs, *L* = 0, *λ* = 710 nm; (**c**) pulsed LG beam with *τ* = 100 fs, *w*_0_ = 100 μm, *λ* = 795 nm.

In Fig. 3 the dependences of the arrival times of BG pulsed beams relative to a plane-wave pulse on the cone angle *θ* (a), the central peak width (beam radius) *w*_*B*_, and orbital angular momentum *L* at the propagation distance *z* = 1 m are presented. The pulse duration *τ* = 100 fs, and the wavelength *λ* = 710 nm.

It follows from the calculations that a decrease in the velocity compared to the velocity of light *c* leads to a delay of $\delta {z}_{B}\approx \frac{z}{2}{\theta }^{2}\approx \frac{z}{2}\frac{{\lambda }^{2}}{{w}_{B}^{2}}$, where $\theta =si{n}^{-1}(\alpha /k)$, ${w}_{B}\approx 2.4/(k\,\sin \,\theta )$ is the central peak width of a Bessel beam, and $k=\frac{2\pi }{\lambda }$, where *λ* is the central wavelength. Consequently, for the time delay and the velocity reduction, we obtain $\delta T\approx (\frac{z}{c})\frac{{\lambda }^{2}}{{w}_{B}^{2}}$ and $\delta v\approx c\frac{{\lambda }^{2}}{{w}_{B}^{2}}$.

These results are in good agreement with the experimental data^22,23^. The time delays presented in Fig. 3a agree well with the measured delays for the Bessel beam in^22^. In^22^, it was reported that the group delay increases with the square of the diameter of the Gaussian beam. Here we have shown that the delay time increases as the diameter of the incident beam decreases. However, there is no contradiction between these results. The point is that the incident beam in^22^ does not propagate in free space, but it is focused by the lens. The spot size of the focused beam that is responsible for the delay is inversely proportional to the waist of the input beam: ${w}_{0}=f\lambda /{w}_{in}$, where *f* is the focal length of the lens, and *w*_*in*_ is the waist of the input beam. Therefore, the larger the diameter of the input beam, the smaller the waist of the focused beam. The calculated delay times depending on OAM are also in good agreement with the measurements in^23^, if the paraxial beams with *w*_0_ >> *λ* are considered.

**Figure 4:**
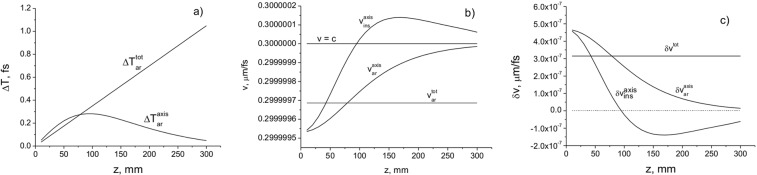
The delays in arrival time *δT* (**a**), the velocities $v=c-\delta v$ (**b**), and the velocity changes *δv* (**c**) as a function of the propagation distance *z*. The beam parameters are: *a*_0_ = 200 μm; *w*_*B*_ = 200 μm; *λ* = 710 nm.

In Fig. 4a the delay times $\delta {T}^{axis}={T}_{ar}^{axis}-{T}_{0}$ and $\delta {T}^{tot}={T}_{ar}^{tot}-{T}_{0}$, where ${T}_{0}=\frac{z}{c}$, depending on the distance *z*, are presented for the pulsed BG beam. The delay of the centre of gravity of the entire pulse beam relative to the plane wave increases linearly with the distance (Fig. 4a). However, the delay of the centre of gravity of the axial part of the pulse beam increases only in the region close to the source, and it disappears with increasing propagation distance. In Fig. 4b the on-axis ${v}_{ar}^{axis}$, ${v}_{ins}^{axis}$, and total cross-section ${v}_{ar}^{tot}$ velocities are presented. The total cross-section velocity depends on the initial parameters of the pulsed beam and remains constant during propagation (Fig. 4b,c).

**Figure 5:**
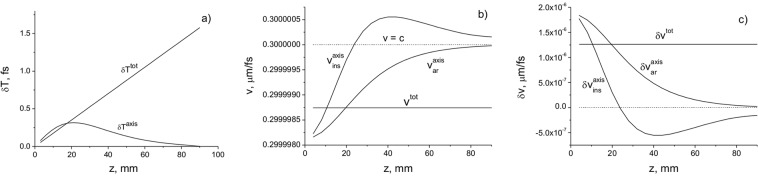
Delays in arrival time *δT* (**a**), the velocities *v* = *c* − *δv* (**b**), and the velocity changes *δv* (**c**) as a function of the propagation distance *z* for a pulsed BG beam with the parameters: *a*_0_ = 100 μm; ${w}_{B}=2.405/(k\,\sin \,\theta )$ = 100 μm, where *θ* is the cone angle; *λ* = 710 nm.

It can be seen that the total beam cross-section velocity ${v}_{ar}^{tot}$ is less than *c* for spatially structured pulsed beams. However, the on-axis velocity determined on the optical axis at a single point varies with the distance from the source. In this case the instantaneous on-axis velocity ${v}_{ins}^{axis}$ can be higher than *c*, even though the velocity ${v}_{ar}^{axis}$ is subluminal (Fig. 4b). Indeed, it follows from the calculation that the instantaneous on-axis velocity of the pulse becomes superluminal in the Rayleigh diffraction region. The superluminality gradually disappears with distance due to the vanishing of the interference term in (8) (Fig. 4b,c). This indicates that superluminal propagation is due to interference between different modes (second term in (25)). For $z\gg {l}_{d}$, the average velocity which is estimated as ${\bar{v}}_{ins}^{axis}=(\frac{1}{d}){\int }_{0}^{d}{v}_{ins}^{axis}(z)dz$, where *T*_*ar*_ is the time of arrival of the pulse centre of gravity, is less than *c* (Fig. 4b). The velocity difference $\delta {v}_{ar}^{axis}=c-{v}_{ar}^{axis}$decreases, approaching zero with increasing distance (Fig. 4c). The on-axis velocity of the centre of gravity ${v}_{axis}=c-\delta {v}_{axis}$ increases with distance *d*, since the value $\delta {v}_{axis}$ decreases with distance (Fig. 4). When *z* → ∞, the velocity difference $\delta {v}_{axis}\to 0$, thus, the on-axis velocity ${v}_{axis}\to c$. Note that the distance region, where the instantaneous on-axis velocity is higher than *c*, decreases with the decrease in the beam cross-section radius (see Fig. 5).

**Figure 6:**
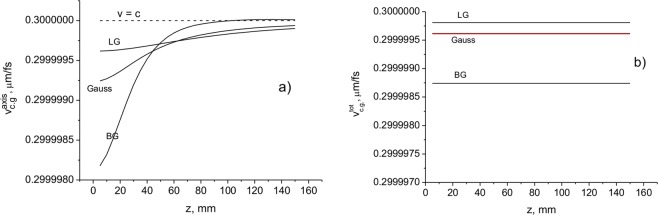
On-axis (**a**) and total cross-section (**b**) velocities *v* = *c* − *δv* as a function of propagation distance for BG, LG, and Gauss pulsed beams. The beam parameters are: BG − *a*_0_ = 100 μm; *w*_*B*_ = 100 μm; LG − *w*_0_ = 100 μm, *w*_0_ = 70 μm; Gauss − *a*_0_ = 100 μm. *λ* = 710 nm.

In Fig. 6, the delay times and velocities as a function of distance *z* for the pulsed BG, LG and Gauss beams are presented. It can be seen that the velocities ${v}_{ar}^{axis}$ and ${v}_{ar}^{tot}$ in the region close to the source are lower for the BG beam than for the LG and Gauss beams with the same beam waists. Differences in the speed of light on the order of 10^−5^–10^−6^ *c* can be observed for the considered pulsed beams.

**Figure 7:**
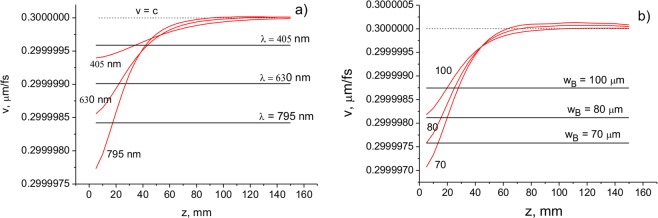
Velocities ${v}_{ar}^{axis}$ (red lines) and ${v}_{ar}^{tot}$ (black lines) as a function of distance for different values of the wavelength (**a**) and central peak width (cone angle) (**b**) of a Bessel-Gauss beam. The beam parameters are: (**a**) *a*_0_ = 100 μm, *w*_*B*_ = 100 μm; (**b**) *a*_0_ = 100 μm; *λ* = 710 nm.

It can be seen that the on-axis velocity varies with the distance approaching the value of *c* at $z > {l}_{d}=\frac{k{w}_{0}^{2}}{2}$, where *l*_d_ is the Rayleigh diffraction length. The total cross-section velocity ${v}_{ar}^{tot}$ decreases with wavelength and remains constant throughout the propagation distance. The velocity ${v}_{ar}^{tot}$ decreases with the decrease in the beam waist and does not change with distance. In Fig. 7, the velocities ${v}_{ar}^{axis}$ and ${v}_{ar}^{tot}$ as a function of the distance for different values of the wavelength and beam waist are presented. It can be seen that the on-axis velocity varies with the distance, approaching the value of *c* at $z > {l}_{d}=\frac{k{w}_{0}^{2}}{2}$, where *l*_*d*_ is the Rayleigh diffraction length. The total cross-section velocity ${v}_{ar}^{tot}$ decreases with the wavelength and remains constant throughout the propagation distance (Fig. 7a,b). The velocity ${v}_{ar}^{tot}$ decreases with the decrease in the beam waist and does not change with distance (Fig. 7a,b).

The spatial delays can be approximated by the expression $\delta z\approx \frac{z{\lambda }^{2}}{{w}_{0}^{2}}$. Consequently, for the time delay and the velocity reduction, we obtain $\delta T\approx (\frac{z}{c})\frac{{\lambda }^{2}}{{w}_{0}^{2}}$ and $\delta v\approx c\frac{{\lambda }^{2}}{{w}_{0}^{2}}$. Note that the delays increase as the wavelength increases and the beam width decreases. When ${w}_{0}\to \infty$, $\delta v\to 0$; therefore, there is no delay of the pulse relative to the plane-wave case. Similarly, $\delta v\to 0$, when $\lambda \to 0$. These results indicate that short wavelengths propagate with a speed close to the plane wave velocity *c*, and long wavelengths undergo a delay.

**Figure 8:**
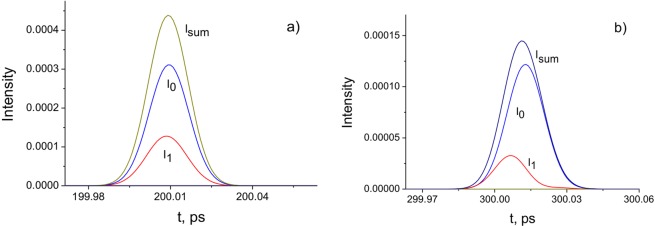
On-axis intensities of BG pulse beam corresponding to the terms *I*_0_ and *I*_1_ in (26): (**a**) *z* = 60 mm; (**b**) *z* = 90 mm. *a*_0_ = 100 μm; *w*_*B*_ = 30 μm; *τ* = 10 fs; *λ* = 710 nm.

In Fig. 8, the pulse shapes on the propagation axis (*ρ* = 0) corresponding to the terms *I*_0_ and *I*_1_ in (26) and their sum are presented for the BG pulse beam. It is shown that the pulse associated with the interference term *I*_1_ propagates at a higher rate than the pulse corresponding to the term *I*_0_. Therefore, in this case, superluminal behaviour may be observed. This result indicates that the local pulse velocity may be higher than *c*. However, the average velocity, which is estimated by the time of arrival of the pulse at $z > {l}_{d}$, is less than *c*.

## Discussion

Variation of the propagation velocity with distance is due to the interference of propagating modes; thus, the superluminal and subliminal behaviours can be observed.

Note that the arrival time and velocity of the pulse beam depend on the measurement method (size and position of the receiver’s aperture). The central and periphery parts of the beam propagate at different velocities; therefore, depending on the size of the aperture of the photodetector and its position in space, the velocities will be different. When only a part of the beam cross-section is registered (for example, at *ρ* = 0), both the phase and group velocities of the modes affect the pulse velocity. Since the phase velocities of the modes exceed the velocity of light *с*, a superluminal propagation of the pulse can be observed near the source, i.e., the pulse propagates over small distances faster than the speed of light in vacuum. However, the average velocity determined by the arrival time of the pulse at $z > {l}_{d}$ is less than *c*, even though the pulse beam experiences superluminal behaviour at some part of the propagation distance.

Given that the entire beam cross-section (total pulse power) has been recorded, averaging over the beam cross-section occurs. In this case, the pulse velocity is determined only by the group velocities of the modes, i.e., the measured pulse velocity will be less than the speed of light *с*.

Note that the energy velocity is always less than *c*, since it is determined only by the group velocities of the propagating modes.

**Figure 9:**
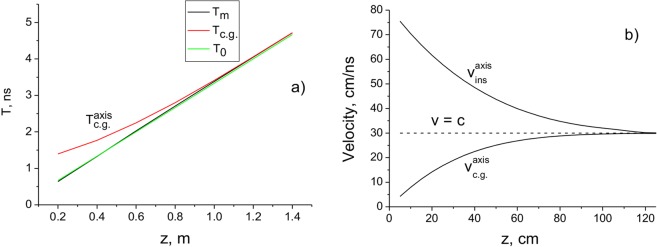
(**a**) Arrival times T_c.g._ (red – pulse centre of gravity), T_m_ (black – pulse maximum), T_0_ = *z/c* (green – light speed *c*) of the microwave pulse (**a**) and velocities (**b**) as a function of distance *z*. *τ* = 2 ns; *λ* = 3.5 cm.

Superluminal behaviour can also be caused by the proximity of the receiving antenna to the emitter, i.e., when the distance between the emitter and receiver $d < c\tau$. It follows from the simulation that the centre of gravity of the pulsed beam exhibits significant superluminal behaviour throughout the propagation. This effect was apparently observed experimentally in^34^, where a noticeable superluminality for *z* < 1 m was detected. In Fig. 9, the delay times of a microwave pulse with a duration of 2 *ns* depending on the distance *z* are presented. The angular frequency of the carrier is 8.6 GHz (*λ* = 3.5 cm). As shown, that the time delay determined by the time of arrival of the pulse amplitude is almost the same as that determined by the speed of light *c* for all distances. However, the time delay of the pulse centre of gravity differs significantly from the one that is defined by the arrival time of the pulse amplitude for a distance of 1.4 *m*. The instantaneous (punctual) velocity of the pulse centre of gravity in this region is higher than *c*, i.e., superluminal behaviour can be observed. This result is in good agreement with the data of^34^, where superluminal behaviour during microwave propagation was observed experimentally. Note that there is no such superluminality in the propagation velocity of the pulse amplitude (Fig. 9).

**Figure 10:**
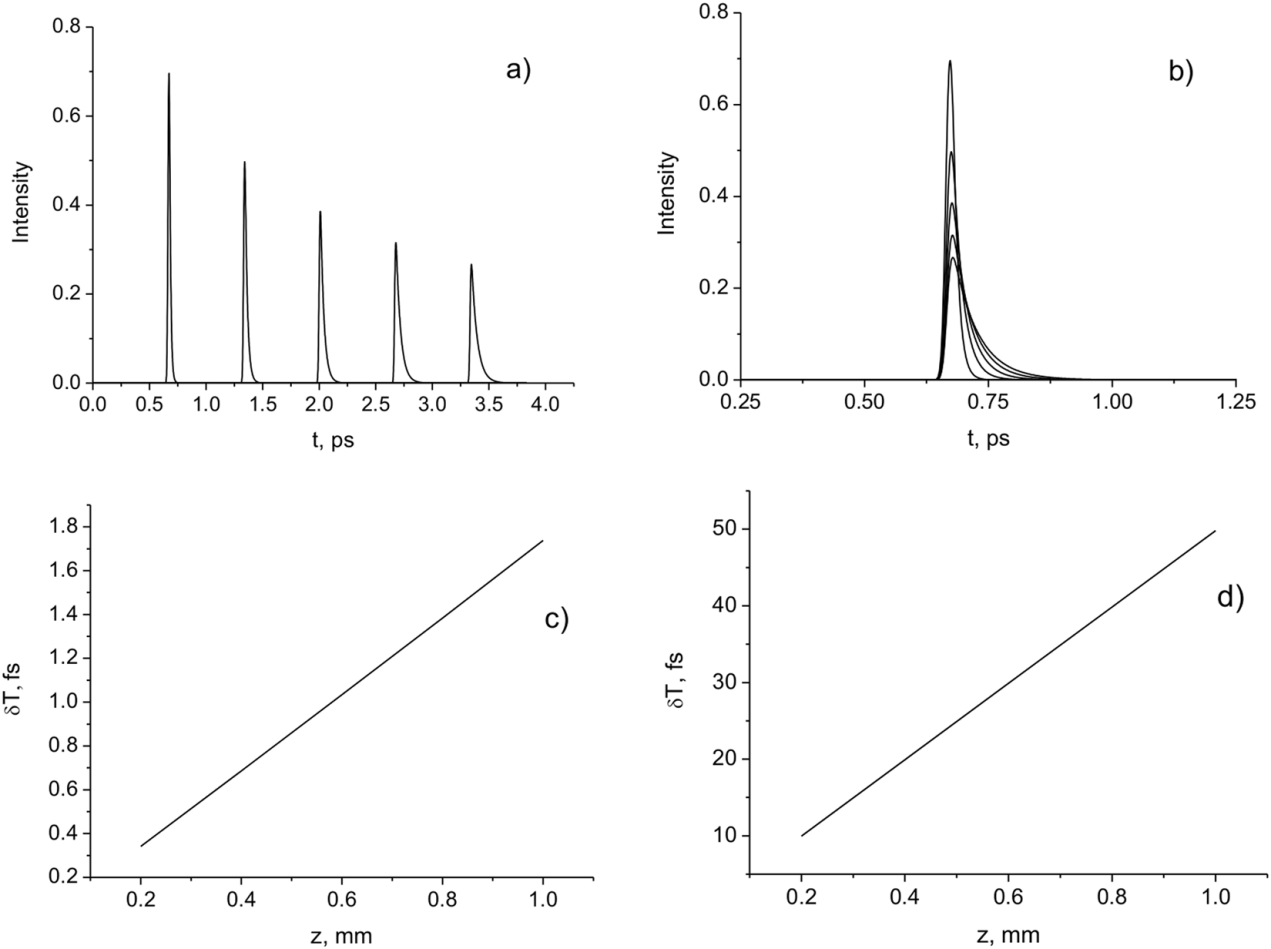
(**a**) Pulse intensities at the distances *z*_k_ = 200, 400, 600, 800, 1000 μm; (**b**) Pulse intensities in the coordinate system with time delay $t-\frac{({z}_{k}-{z}_{1})}{c}$; (**c**,**d**) Delays in arrival time as a function of distance *z*. *a*_0_ = 1 μm; *w*_*B*_= 2 μm; *τ* = 10 fs; *λ* = 630 nm.

The dispersion effects become significant for strongly focused pulsed beams. It is known that in this case nonparaxial effects become noticeable^35,36^. In this case, the influence of the longitudinal field component *E*_*z*_ and the spin-orbit interaction should also be taken into account^37,38^. In Fig. 10a,b, the BG pulse intensities at different distances z_k_ are presented. In Fig. 10b, the pulses are presented in an offset time scale $t-\frac{({z}_{k}-{z}_{1})}{c}$, where *z*_*k*_ is the distance, at which the pulse is recorded. In Fig. 10c,d, the delay times of the pulsed BG beam (the arrival times of the energy ${T}_{E}=z/{v}_{E}$ (c) and pulse centre of gravity ${T}_{ar}^{tot}$ (d) compared to light in vacuum), depending on the distance *z*, are presented.

It is shown that the pulse shape changes during propagation due to dispersion effects. Note that the beam width at the front of the pulse changes less than that at the tail part during the propagation. This observation indicates that the frontal part (high frequencies) of the pulse is less affected by the diffraction. In^2^, this effect is linked with the existence of a time-dependent “vacuum dispersion”. The pulse front can be called a “diffractive precursor”, since diffraction effects mainly develop behind it. As a result, the problem of the diffraction of the pulsed beams becomes analogous to Sommerfeld and Brillouin’s problem of the propagation of a signal with a sharp leading edge in a medium with a time-dependent dispersion^39,40^. The front part (precursor) has a small amplitude and a high frequency. The main part of the signal (the signal body) always propagates at a speed less than *c*. This part of the signal is usually recorded by the detector. As the sensitivity of the detector increases, the speed obtained from the measurements will approach speed *c*.

The slowing down of the light propagation velocity in waveguides is well known and can be explained by the delays of modes. In^41^, it is shown that at this delay also depends on the magnitude of the orbital angular momentum (helicity of the wave front) and the spin angular momentum (polarization) of the propagating beam. Therefore, it can be assumed that a polarization similar to the orbital angular momentum will affect the propagation velocity in the free space of pulsed beams with OAM and SAM. A detailed analysis of these effects will be presented in future work.

The obtained results do not contradict the experimental data obtained for femtosecond, picosecond, and nanosecond time-domain pulses in the visible and THz regions.

A number of effects have been discovered where light travels over small distances faster than the speed of light in vacuum. In this regard, the question arises whether it is possible to exchange information superluminally^42,43^. As emphasized in^43^, the information content of the signal tends to decrease during superluminal propagation due to noise and for this reason superluminal information exchange is impossible. Here, it is shown that the superluminal signal can be obtained only for the axial part of the incident pulsed beam. Only an axial point on the cross-section of the pulsed beam can propagate for short distances at a velocity *v* > *c*. The entire pulse beam always propagates at *v* < *c*. Hence, the information (for example, an image encoded with spatial beams) carried by the entire pulse beam can be transmitted only subluminally. Indeed, a single pixel of the image does not transmit information about the entire image, or the image information cannot be decoded from a point (pixel) on the receiver plane. Despite the fact that individual pixels can be transmitted superluminally, they do not contain all of the original information. If the receiver detects a total incident pulse beam, superluminal signal transmission does not occur.

It was experimentally established that single photons travel at the group velocity^44^. Recently, it was shown that, depending on the measurement scheme, the group velocity can be defined differently^45^. Different versions of the group velocity of the Bessel-Gauss pulses have been considered, depending on how the pulse is recorded in the output plane, namely, integrally or with spatial resolution. In^22^, it is shown that transverse structuring of a photon results in a decrease in the group velocity along the axis of propagation. It has now been shown that the on-axis part of arbitrary pulsed beams propagates at variable speeds, exhibiting both superluminal and subluminal behaviours. The effect comes from a rigorous calculation of the impulse evolution.

Usually, the terms “group” and “phase” velocities are used to describe the pulse velocity. However, these velocities are well defined only for plane waves. In the case of spatially structured pulsed beams, the term “impulse velocity” is more appropriate (see also^46^). Indeed, the impulse velocity incorporates the group and phase velocities of a set of spatial mode components.

The superluminal effect is due to interference between different modes (second term in (26)). The interference terms disappear due to the orthogonality of the modal functions if integration across the entire beam cross-section is taken. Therefore, the superluminal effect can be observed if only the pulsed beam electric field is measured at one point in the transverse plane (for example, *ρ* = 0). There is no superluminal effect if the entire cross-section of the pulse beam is recorded.

## Methods

The Maxwell equations describing the propagation of light are reduced to

1 $${\nabla }\times {\nabla }\times \overrightarrow{E}=-\,\frac{1}{{c}^{2}}\frac{{\partial }^{2}\overrightarrow{E}}{\partial {t}^{2}}$$

To solve Eq. (1), let us make the following simplifying assumptions. We assume that the considered field is quasi-monochromatic, i.e., the spectrum with a central frequency *ω*_0_ has a width *Δω* such that $\varDelta \omega /\omega \ll 1$. This is valid for most sources of radiation used in the experiments.

It is possible to separate a rapidly changing part of the electric field using the approximation of slowly changing amplitudes:

2 $$\overrightarrow{E}(\overrightarrow{r},t)=\frac{1}{2}\overrightarrow{x}[E(\overrightarrow{r},t)\,{\exp }(\,-\,i{\omega }_{0}t)+conj.],$$

where $E(\overrightarrow{r},t)$ is the slowly changing function of time (relative to the period of the optical wave), $\overrightarrow{r}=(x,y,z)$.

Substituting (2) into Eq. (1), we find that the Fourier components $E(\overrightarrow{r},\omega -{\omega }_{0})$, defined as

3 $$E(\overrightarrow{r},\omega -{\omega }_{0})={\int }_{-\infty }^{\infty }E(\overrightarrow{r},t)\,{\exp }[i(\omega -{\omega }_{0})t]dt,$$

satisfy the Helmholtz equation

4 $${{\nabla }}^{2}\overrightarrow{E}+{k}^{2}\overrightarrow{E}=0,$$

where *k* = *ω*/*c* is the wavenumber.

There are exact solutions to the Helmholtz equation in the form of Bessel modes or non-diffracting beams^32,33,47,48^:

5 $$J(\alpha ,\rho ,z,\omega )={J}_{l}(\alpha ,\rho )\,{\exp }\{i\beta (\omega )z\},$$

where ${J}_{l}(\alpha ,\rho )$ is the Bessel function of the first kind, $\alpha =k\,\sin \,\theta$, *θ* is the cone angle of the Bessel beam, *l* is the azimuthal index corresponding to OAM, and *β* is the propagation constant.

Each spectral component propagates independently according to Eq. (4). Note that the Bessel beams have infinite transverse size and require infinite power. In practice, the quasi-Bessel beams of bounded transverse dimensions, which are generated by an axicon or conical lens, are used. These beams exhibit no diffraction over a limited propagation distance^32,33^. This property of Bessel beams allows for useful applications in various areas such as three-dimensional imaging^49^, nonlinear optics^50^, dispersion compensation^51^, and wireless communication^52^.

Consider the incident pulsed beam $E(\rho ,0,t)={A}_{0}\psi (\rho )f(t)$ in the plane *z* = 0, the envelope of which is described by the function $f(t)={\exp }(-\,{t}^{2}/2{\tau }^{2}+i{\omega }_{0}t)$, where *τ* is the input pulse width.

The frequency spectrum of this signal is determined by the expression

6 $$F(\omega -{\omega }_{0})=(1/\sqrt{2\pi }){\int }_{-\infty }^{\infty }f(t){e}^{-i\omega t}dt=(\frac{\tau }{\sqrt{2\pi }}){\exp }[-{(\omega -{\omega }_{0})}^{2}{\tau }^{2}/2].$$

The evolution of the spatial distribution of the field for a given spectral frequency component is determined by

7 $$E(\rho ,z,\omega -{\omega }_{0})={\int }_{0}^{{\alpha }_{max}}\alpha {J}_{l}(\alpha \rho ){c}_{l}(\alpha )F(\omega -{\omega }_{0})\exp (i\beta (\omega )z)d\alpha ,$$

where ${c}_{l}(\alpha )={\int }_{0}^{\infty }\rho {J}_{l}(\alpha \rho )\psi (\rho )d\rho$, *F*(*ω*) is the frequency spectrum of the incident pulse, and $\beta ={({k}^{2}-{\alpha }^{2})}^{\frac{1}{2}}$.

Decomposition (7) is called the Fourier-Bessel integral (see, for example^48^).

Substituting (6) into (7), we obtain

8 $$E(\rho ,z,\omega -{\omega }_{0})={\int }_{0}^{{\alpha }_{max}}\alpha {J}_{l}(\alpha \rho ){c}_{l}(\alpha )\exp [-\frac{{(\omega -{\omega }_{0})}^{2}{\tau }^{2}}{2}]\exp (i\beta (\omega )z)d\alpha $$

The inverse Fourier transform of (7) gives an expression for the electric field in the time domain:

9 $$E(\rho ,z,t)=\frac{1}{2\pi }{\int }_{-\infty }^{\infty }E(\rho ,z,\omega -{\omega }_{0}){\exp }[-i(\omega -{\omega }_{0})t]d\omega .$$

Expand *β*(*ω*) in a Taylor series in the neighbourhood of *ω*_0_:

10 $$\beta (\omega )=\sum _{m=0}\frac{{(\omega -{\omega }_{0})}^{m}}{m!}{\gamma }_{m}={\gamma }_{0}+(\omega -{\omega }_{0}){\gamma }_{1}+\frac{{(\omega -{\omega }_{0})}^{2}}{2!}{\gamma }_{2}+\ldots ,$$

where $${\gamma }_{m}=\frac{{d}^{m}}{d{\omega }^{m}}\beta (\omega )|\omega ={\omega }_{0}$$, $${\gamma }_{1}=\frac{d\beta }{d\omega }=\frac{1}{c}\frac{d\beta }{dk}$$.

Substituting (8) into (9), we obtain

11 $$E(\rho ,z,t)=\frac{\tau }{2\pi }{\int }_{0}^{{\alpha }_{max}}\alpha {c}_{l}(\alpha ){J}_{l}(\alpha \rho )\exp (i\beta ({\omega }_{0})z)f(t,z,\tau )d\alpha ,$$

where $$f(t,z,\tau )=\sqrt{\frac{2\pi }{{\tau }^{2}-iz{\gamma }_{2}}}{\exp }[-\frac{{(t-z{\gamma }_{1})}^{2}}{2({\tau }^{2}-iz{\gamma }_{2})}]$$.

There are also modal solutions of finite transverse dimensions with discrete azimuthal and radial indices similar to the modes in cylindrical waveguides. The transverse field profiles of these solutions remain invariant along the effective depth of field (diffraction-free region), i.e., exhibit modal properties. Note that these solutions form a complete set of mutually orthogonal functions in a given interval [0, *R*_0_]. Hence, any field in the initial plane *z* = 0 can be decomposed into these modal solutions.

The normalized Bessel functions with radial *p* and azimuthal *l* indices can be considered as the modal solutions of Eq. (4) within the effective depth of field:

12 $${\psi }_{pl}(\rho ,\varphi )=\frac{{J}_{l}({\mu }_{pl}\frac{\rho }{{R}_{0}}){\exp }(il\varphi )}{(\sqrt{\pi }{R}_{0}{J}_{l+1}({\mu }_{pl}))},$$

where ${\mu }_{1},{\mu }_{2},\ldots$ are the positive zeros of the Bessel function ${J}_{l}(z)$.

It follows from the orthogonality condition for Bessel functions^47,48,53,54^

13 $${\int }_{0}^{{R}_{0}}{J}_{m}({\mu }_{i}\rho /{R}_{0}){J}_{m}({\mu }_{j}\rho /{R}_{0})\rho d\rho =\frac{{R}_{0}^{2}}{2}{[{J}_{m+1}({\mu }_{i})]}^{2}{\delta }_{ij},$$

that these modes satisfy the equation

$${\int }_{0}^{2\pi }{\int }_{0}^{{R}_{0}}{\psi }_{pl}^{\ast }(\rho ,\varphi ){\psi }_{pl}(\rho ,\varphi )\rho d\rho d\varphi =1.$$

Analogously to the Fourier-Bessel expansion^47,48,53,54^

$$f(r,0)=\mathop{\sum }\limits_{k=1}^{\infty }{c}_{k}{J}_{m}({\mu }_{k}r),$$

where ${c}_{k}=\frac{2}{{R}_{0}^{2}{[{J}_{m+1}({\mu }_{k})]}^{2}}{\int }_{0}^{{R}_{0}}tf(t){J}_{m}({\mu }_{k}t)dt,$an arbitrary electric field at *z* = 0 can be expanded in the series of these discrete modes:

14 $$E(\rho ,0,\omega )=\mathop{\sum }\limits_{k=1}^{\infty }{c}_{k}{J}_{m}({\mu }_{k}\frac{\rho }{{R}_{0}}),$$

where ${\mu }_{1},{\mu }_{2},\ldots$ are the positive zeros of the function *J*_*m*_(*z*),

$${c}_{k}=(\frac{2}{({R}_{0}^{2}{[{J}_{m+1}({\mu }_{k})]}^{2})}){\int }_{0}^{{R}_{0}}\rho f(\rho ){J}_{m}({\mu }_{k}\rho /{R}_{0})d\rho .$$

Note that in^55^, this decomposition of the field was used when considering the scattering of light by small particles.

**Figure 11:**
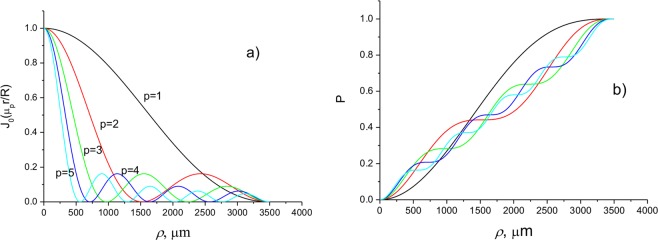
Intensities of normalized Bessel modes (**a**) and powers of Bessel modes (**b**) with different radial indices *p* and zero azimuthal index *l* = 0 as a function of the transverse coordinate *ρ*.

In Fig. 11a, the normalized Bessel mode intensities (12) with OAM *l* = 0 and different radial indices *p* as a function of the transverse coordinate are presented. In Fig. 11b, the powers of the Bessel modes as a function of the radial distance *ρ* are presented. In contrast to Bessel beams, the considered Bessel beams are shown to have finite transverse size and finite power.

The evolution of the electric field is determined by the expression

15 $$E(\rho ,z,\omega -{\omega }_{0})=F(\omega -{\omega }_{0})\sum _{pl}{c}_{pl}{\psi }_{pl}(\rho ){\exp }(i{\beta }_{pl}(\omega )z),$$

where *c*_*pl*_ are the modal coefficients depending on the incident field, and ${\beta }_{pl}(\omega )=k{(1-{(\frac{{\mu }_{pl}}{(k{R}_{0})})}^{2})}^{\frac{1}{2}}$ is the propagation constant of the mode with radial *p* and azimuthal *l* indices, respectively.

The coefficients *c*_*pl*_ are determined by the incident pulse field $E(\rho ,0,t)=E(\rho ,0)f(t)$:

16 $${c}_{pl}={\int }_{0}^{\infty }{\int }_{0}^{2\pi }E(\rho ,0){\psi }_{pl}^{\ast }(r,\varphi )\rho d\rho d\varphi .$$

Substituting the frequency spectrum (8) into (15), it can be obtained that

17 $$E(\rho ,z,\omega -{\omega }_{0})=(\frac{\tau }{\sqrt{2\pi }})\mathop{\sum }\limits_{p=1}^{N}{c}_{pl}{\psi }_{pl}(\rho ){\exp }(i{\beta }_{pl}z){\exp }[-\frac{{(\omega -{\omega }_{0})}^{2}{\tau }^{2}}{2}]$$

The inverse Fourier transform of (17) gives an expression for the electric field in the time domain:

18 $$E(\rho ,z,t)=(\frac{\tau }{\sqrt{2\pi }})\mathop{\sum }\limits_{p=1}^{N}{c}_{pl}{\psi }_{pl}f(t,z,\tau ),$$

where $$f(t,z,\tau )=\frac{{\exp }[i(t-{\gamma }_{0,p}z)]}{{({\tau }^{2}+i{\gamma }_{2,p}z)}^{\frac{1}{2}}}{\exp }\{-\frac{{(t-{\gamma }_{1,p}z)}^{2}}{2({\tau }^{2}+i{\gamma }_{2,p}z)}\}.$$

It can be seen from (18) that the electric field of each mode reaches its maximum value at

$$t=z{\gamma }_{1}=z\frac{d\beta }{d\omega }=\frac{z}{c}\frac{d\beta }{dk},$$

where ${v}_{g}=c{(\frac{d\beta }{dk})}^{-1}=c{[1-{\mu }_{pl}^{2}/({R}^{2}{k}^{2})]}^{\frac{1}{2}}$ is the group velocity of the mode.

The higher the mode number (radial *p* and azimuthal *l* indices) or larger the transverse wave number is, the lower the group velocity. This result is consistent with the natural waveguide idea discussed in^28^, where the slowing of a Bessel light beam is shown with the increase in the transverse wave number. Recently, a differential group delay of the space-time wave packet in free space of approximately 150 ps was recorded^20^. These order group delays can be observed if high-order modes are used.

The group velocity of the mode acquires a maximum value of *c* when *λ* → 0, which corresponds to the approximation of geometric optics.

The phase velocity of the mode is equal to:

19 $${v}_{ph}=\frac{\omega }{\beta }=c{[1-{\mu }_{pl}^{2}/({R}^{2}{k}^{2})]}^{\frac{-1}{2}}.$$

It can be seen from above, that ${v}_{g} < c$, ${v}_{ph} > c\,$and ${v}_{g}{v}_{ph}={c}^{2}$. The higher the mode number is, the greater the phase velocity. However, this does not mean that energy or information can be transmitted at this rate.

The term $\tau (z)={\tau }_{0}{(1+\frac{{\gamma }_{2,p}^{2}{z}^{2}}{{\tau }_{0}^{4}})}^{\frac{1}{2}}$ defines the change in pulse width due to modal dispersion.

**Figure 12:**
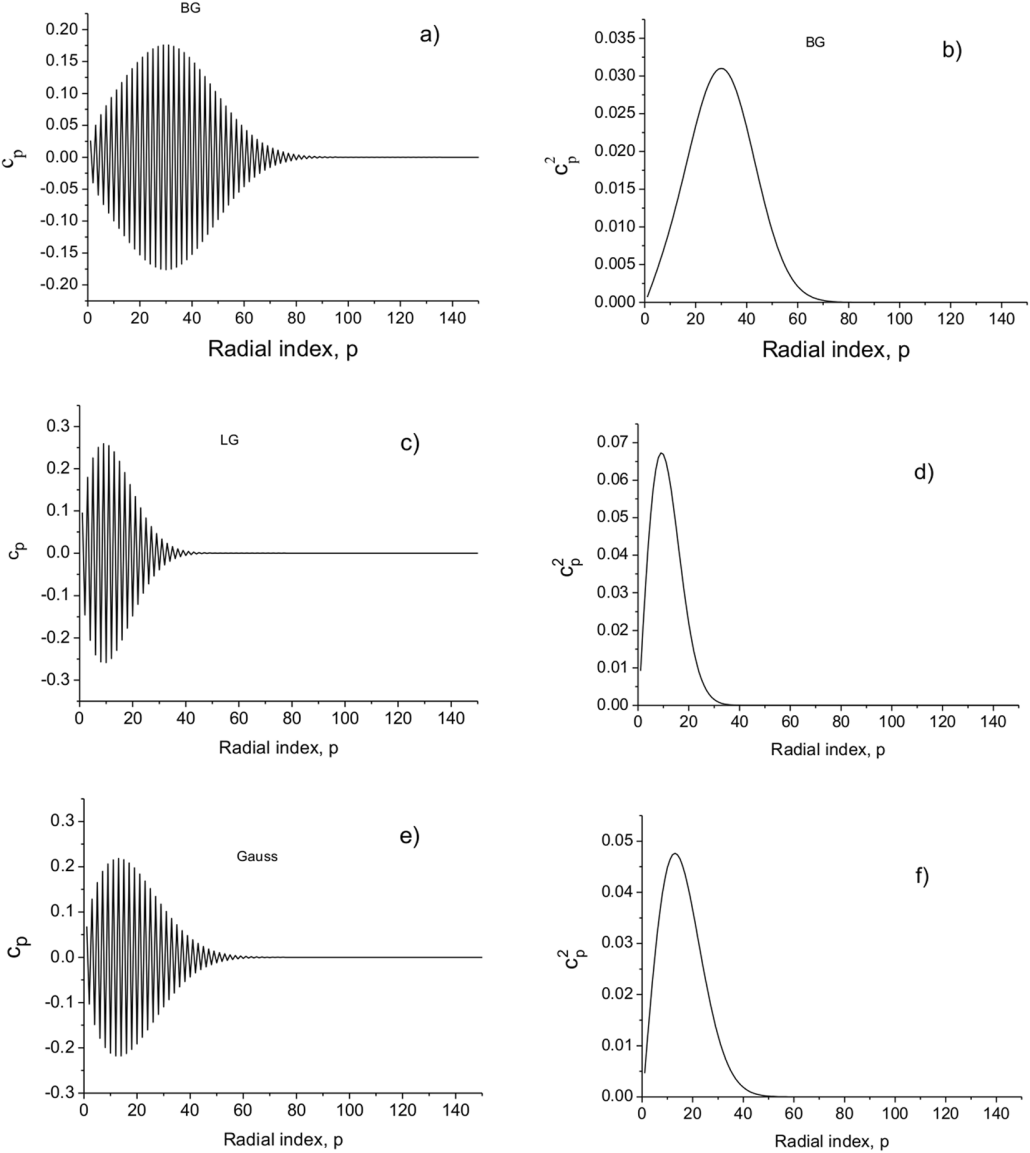
Modal coefficients *c*_*pl*_ (**a**,**c**,**e**) and their squares (**b**,**d**,**f**) as a function of the radial index *p*: (**a**,**b**) BG beam; (**c**, **d**) LG beam; (**e**,**f**) Gauss beam. *a*_0_ = 50 μm, *w*_*B*_ = 50 μm.

A limited number of modes *N* can be taken in summation (18). In Fig. 12, the coefficients *c*_*pl*_ as a function of the radial mode number are presented for the pulsed BG beam. It is shown that a limited number of propagating modes are excited in free space. This greatly simplifies computations compared to the integration using (11).

Note that the intensity distributions along the radius of the Bessel-Gauss beam in different planes perpendicular to the beam axis obtained by modal decomposition (17) are in good agreement with the results of numerical calculations obtained using the Fresnel diffraction integral^56^. For Bessel-Gauss beams the diffraction spreading is significantly less than for Gaussian beams, i.e., these beams have a sharp radiation pattern.

Consider the incident pulsed beams at *z* = 0 with Gaussian, Bessel-Gauss, and Laguerre-Gauss spatial distributions, respectively:

20 $$E(\rho ,0,t)={A}_{0}{\exp }\,(\,-\,{\rho }^{2}/{a}_{0}^{2})f(t);$$

21 $$E(\rho ,0,t)={A}_{0}{\exp }(\,-\,{\rho }^{2}/{a}_{0}^{2}){J}_{l}(\alpha \rho ){\exp }(il\varphi )f(t);$$

22 $$E(\rho ,0,t)={A}_{0}{\psi }_{pl}(\rho ,\varphi )f(t);$$

where *a*_0_ is the Gaussian beam radius,

$${\psi }_{pl}(\rho ,\varphi )={(\frac{1}{\pi {w}_{0}^{2}})}^{\frac{1}{2}}{[\frac{p!}{(p+l)!}]}^{\frac{1}{2}}{({\rho }^{2}/{w}_{0}^{2})}^{\frac{l}{2}}\exp (-\,\frac{{\rho }^{2}}{2{w}_{0}^{2}}){L}_{p}^{l}({\rho }^{2}/{w}_{0}^{2})\exp (il\varphi ).$$

The integrals (16) for the considered beam pulses can be calculated analytically^57^. The following expressions for modal coefficients are obtained for Gaussian, BG and LG pulsed beams, respectively:

23 $${c}_{p}=\frac{{A}_{0}\sqrt{2}{a}_{0}}{{R}_{0}{J}_{1}({\mu }_{p})}{\exp }\{-\frac{{a}_{0}^{2}{\mu }_{p}^{2}}{4{R}_{0}^{2}}\};$$

24 $${c}_{pl}=\frac{{A}_{0}{a}_{0}^{2}}{\sqrt{\pi }{R}_{0}{J}_{l+1}({\mu }_{p}{R}_{0})}{\exp }[-({\alpha }^{2}+{\mu }_{p}^{2}){a}_{0}^{2}/4]{I}_{l}(\frac{\alpha {\mu }_{p}}{2}{a}_{0}^{2})$$

25 $${c}_{pl}={A}_{0}{B}_{0}{({\mu }_{p}\frac{{w}_{0}}{{R}_{0}})}^{l}{\exp }(-{\mu }_{p}^{2}{w}_{0}^{2}/(2{R}_{0}^{2})){L}_{p}^{l}({\mu }_{p}^{2}{w}_{0}^{2}/{R}_{0}^{2}),$$

where $${B}_{0}=2(\frac{{w}_{0}}{{R}_{0}}){(-1)}^{p}{(\frac{p!}{(p+l)!})}^{\frac{1}{2}}/{J}_{l+1}({\mu }_{p}).$$

The intensity distribution $I(\rho ,z,t)={|E(\rho ,z,t)|}^{2}\,$is determined by the expression

26 $$I(\rho ,z,t)={I}_{0}+{I}_{1}=\frac{1}{2\pi }\mathop{\sum }\limits_{p=1}^{N}{|{c}_{pl}|}^{2}{|{\psi }_{pl}|}^{2}{|{Q}_{pl}|}^{2}+\frac{1}{2\pi }\mathop{\sum }\limits_{p=1}^{N}\mathop{\sum }\limits_{n=1}^{N}{c}_{pl}^{\ast }{c}_{nl}{\psi }_{pl}^{\ast }{\psi }_{nl}{Q}_{pl}^{\ast }{Q}_{nl},\,p\ne n$$

where $${Q}_{pl}=\frac{{\exp }[i(t-{\gamma }_{0,p}z)]}{{({\tau }^{2}+i{\gamma }_{2,p}z)}^{\frac{1}{2}}}{\exp }\{-\frac{{(t-{\gamma }_{1,p}z)}^{2}}{2({\tau }^{2}+i{\gamma }_{2,p}z)}\}.$$

It can be seen, that the first term in (26) is the sum of the intensities of the modes and contains only the group velocities of the modes, while the second term, representing the interference of the modes, includes both the phase ${v}_{ph}=\frac{1}{{\gamma }_{0,p}}$ and group ${v}_{g}=\frac{1}{{\gamma }_{1,p}}$ velocities. This observation indicates that only the second term is responsible for the superluminal effect that occurs due to the interference between modes.

If the integration of intensity across the entire cross-section is taken, then the power (total intensity) *P*(*z*, *t*) is determined by

27 $$P(z,t)=\langle I(\rho ,z,t)\rangle =\frac{1}{2\pi }\sum (\frac{{|{c}_{pl}|}^{2}}{b}){\exp }\{-\frac{{(t-{\gamma }_{1,p}z)}^{2}}{{b}^{2}{\tau }^{2}}\},$$

where $$b={(1+\frac{{\gamma }_{2,p}^{2}{z}^{2}}{{\tau }^{4}})}^{\frac{1}{2}}.$$

The cross-terms in (26) describing the interference between different modes become zero due to the orthogonality condition (13). Consequently, the mode intensity components in (27) reach their maximum values, when $t=z{\gamma }_{1,p}=\frac{z}{{v}_{g}}$, i.e., only the subluminal propagation can be observed by measuring the total intensity over the entire beam cross-section. This result means that the observation of superluminal or subluminal behaviour depends on the method of measurement, i.e., the aperture size of the receiving antenna (the detector).

The pulse velocity at a given point can be determined from Eq. (26). It follows from (26) that both the group and phase velocities of the modes contribute to the resulting speed of the pulse.

The average time of arrival of the centre of gravity of the total beam can be calculated analytically: ${T}_{ar}^{tot}=\langle t\rangle =\mathop{\sum }\limits_{p=1}^{N}{|{c}_{pl}|}^{2}{\gamma }_{1,p}z$. The velocity of the centre of gravity of the total beam is given by ${v}_{c.g.}^{tot}=z/\langle t\rangle$, where ${\gamma }_{1,p}$ is determined by the group velocities of the modes, and hence ${v}_{c.g.}^{tot} < c$. It is clear that this velocity depends on the parameters of the incident pulse beam and remains constant during propagation.

The average width of pulses changes during propagation. The modal dispersion of the pulse beam, defined as ${\sigma }_{\tau }^{2}=\langle {(\varDelta t)}^{2}\rangle =\langle {t}^{2}\rangle -{\langle t\rangle }^{2}$, is given by

28 $${\sigma }_{\tau }^{2}=\mathop{\sum }\limits_{p=1}^{N}{|{c}_{pl}|}^{2}({\gamma }_{1,p}^{2}{z}^{2}+\frac{{\tau }^{2}}{2}+\frac{{\gamma }_{2,p}^{2}{z}^{2}}{(2{\tau }^{2})})-{(\mathop{\sum }\limits_{p=1}^{N}{|{c}_{pl}|}^{2}{\gamma }_{1,p}z)}^{2}.$$

Thus, the pulse width increases with the propagation distance depending on the group velocities, the dispersion of the group velocities, and the pulse duration.

## Conclusions

Thus, the theoretical analysis of the nonparaxial propagation of localized wave packets in free space on the basis of the wave approach is carried out. Fourier-Bessel modes with azimuthal and radial indices are proposed to describe the nonparaxial propagation of arbitrary pulse beams in free space. The modal approach is shown to provide clear physical insight into the subluminal and superluminal behaviours of arbitrary pulsed beams in free space, which arise from the interference of modes and depend on the parameters of the pulsed beam, the aperture of the measuring system and the range of the propagation distance.

The velocities for the pulse amplitude, pulse centre of gravity, and pulse energy flow are considered for Gaussian, Bessel-Gauss, and Laguerre-Gauss pulsed beams.

The importance of the measurement method in the observation of the superluminal effect is emphasized. Usually the spatial dimensions of receivers are smaller than the transverse beam sizes. This indicates that the interference terms (second term in Eq. (26)) will contribute to the velocity of the pulse beam in the measurements. The second term in Eq. (26), representing the interference of the modes, includes both the phase and group velocities of modes. The phase velocities *v*_*ph*_ > *c* (see Eq. (19)). This indicates that only the second term is responsible for the superluminal effect that occurs due to interference between modes. Thus, superluminal propagation occurs due to interference between spatial modes, and its observation is possible if only a part of the beam cross-section is recorded.

It is shown that the pulse beam propagates in free space with variable velocity along the axis. This observation indicates that the average propagation velocity of a pulsed beam with specified initial parameters (pulse duration, beam radius, OAM, and frequency spectrum) depends on the distance between the source and the receiver. The change in propagation velocity along the axis with distance is due to the interference of the propagating modes; hence, superluminal and subliminal behaviours can be observed along the propagation axis. It is shown that the on-axis velocity varies with distance, approaching the fundamental value of *c* at large distances. The velocity of the spatially averaged total pulsed beam is defined by Eq. (27). It is clear that the modal intensity components in Eq. (27) reach their maximum values, when $t=\frac{z}{{v}_{g}}$, i.e., only the subluminal propagation can be observed by measuring the total intensity over the entire beam cross-section. Simulations showed that the velocity of the total pulsed beam is always less than the velocity of the plane wave *c* and remains constant during propagation. As follows from Eqs. (26) and (27), this is true for incident pulsed beams with arbitrary transverse distribution. A similar result was demonstrated in^45^ for the Bessel-Gauss pulses using the concept of a spatially averaged group velocity^22^ and in^58^ for the Gaussian, Bessel-Gauss and Laguerre-Gauss pulsed beams using a rigorous modal approach.

Although the local on-axis velocity of the pulse may be higher than *c*, the average velocity, which is estimated from the arrival time of the pulse at *z* > *l*_*d*_, is less than *c*. The obtained results do not contradict the experimental data on the observation of superluminal and subluminal effects.

The slowing down of the speed of propagation with the decrease in the spatial dimensions of the incident pulsed beam is shown. This phenomenon can be used, for example, in all-optical switching using slow light^59^.

In conclusion, the effect of inconstancy of the pulse beam propagation velocity with distance arises due to the interference of propagating modes; thus, it is possible to observe superluminal and subliminal behaviours. It is shown that the observation of superluminal propagation is possible if only an axial part of the beam cross-section is recorded. Although the decrease in the speed of light for paraxial beams is hardly noticeable, it should be taken into account when accurate distance determination is required. These results are particularly important in applications such as time-of-flight measurements, radio and satellite communications, free-space optical communication, astrophysics, and in quantum information and gravitational wave experiments.
